# Identifying *Schistosoma japonicum* Excretory/Secretory Proteins and Their Interactions with Host Immune System

**DOI:** 10.1371/journal.pone.0023786

**Published:** 2011-08-24

**Authors:** Qi Liao, Xiongying Yuan, Hui Xiao, Changning Liu, Zhiyue Lv, Yi Zhao, Zhongdao Wu

**Affiliations:** 1 Department of Parasitology, Zhongshan School of Medicine, Sun Yat-sen University, Guangzhou, People's Republic of China; 2 Key Laboratory for Tropical Diseases Control, Ministry of Education, Sun Yat-sen University, Guangzhou, People's Republic of China; 3 Bioinformatics Research Group, Key Laboratory of Intelligent Information Processing, Institute of Computing Technology, Chinese Academy of Sciences, Beijing, People's Republic of China; The University of Maryland, United States of America

## Abstract

*Schistosoma japonicum* is a major infectious agent of schistosomiasis. It has been reported that large number of proteins excreted and secreted by *S. japonicum* during its life cycle are important for its infection and survival in definitive hosts. These proteins can be used as ideal candidates for vaccines or drug targets. In this work, we analyzed the protein sequences of *S. japonicum* and found that compared with other proteins in *S. japonicum*, excretory/secretory (ES) proteins are generally longer, more likely to be stable and enzyme, more likely to contain immune-related binding peptides and more likely to be involved in regulation and metabolism processes. Based on the sequence difference between ES and non-ES proteins, we trained a support vector machine (SVM) with much higher accuracy than existing approaches. Using this SVM, we identified 191 new ES proteins in *S. japonicum*, and further predicted 7 potential interactions between these ES proteins and human immune proteins. Our results are useful to understand the pathogenesis of schistosomiasis and can serve as a new resource for vaccine or drug targets discovery for anti-schistosome.

## Introduction

*Schistosoma japonicum* (*S. japonicum*) is one of the major causative agents of schistosomiasis, a tropical parasitic disease that infects more than 200 million people in over 70 countries, and endangers a further 650 million people worldwide [1]. During the life cycle of *S.japonicum*, it is exposed to diverse environmental conditions and changes from an asexual form in the intermediate hosts such as snails to a sexual form in vascular system of the definitive host such as human. It has been reported that large number of proteins excreted or secreted by schistosomas are important for their survival in their hosts [2].

To get through secretory pathways and anchor on the surface of schistosoma, excretory/secretory (ES) proteins (including tegument proteins that are exposed to the host) usually possess certain sequence features. When exposed to host tissues, these proteins not only can stimulate the innate immune system, but also modulate various host immune responses, thus help the pathogen evade the host immune defence and protect them from oxidative stress [2]. For example, the protein *lysophosphatidylserine* in schistosomes can stimulate dendritic cells via Toll-like receptor 2 (*TLR2*) to promote Th2 immune response and regulate T cell development [3]. Therefore, identification of *S.japonicum* ES proteins is important for both understanding parasite host interaction and finding new candidate vaccines, drug targets or diagnostic reagents.

Using mass spectrometry hundreds of ES proteins have been identified in different developmental stages of *S.mansoni* [4], [5], [6], [7], [8] and *S.japonicum* [9], [10], [11]. Despite of that, the number of proteins already found is still very limited due to the capacity of mass spectrometry and *in vitro* culture. Therefore, tools like SignalP [12] and SecretomeP [13] are frequently used to decide whether a protein is secretory. However, these tools are specifically designed for secreted proteins in mammal or bacteria, and may not perform the best in parasite.

In this paper, we compared the sequence features between *S.japonicum* ES proteins collected from literatures [9], [10], [11] (167 proteins, denoted as SjSPs) and background proteins created from NCBI non-redundant protein database (1401 proteins, denoted as SjBgPs) to find out the specific features belonged to ES proteins. Then based on the features of ES and non-ES proteins we trained a SVM model with much higher accuracy than existing approaches. Using this SVM model, we identified 191 new ES proteins in *S.japonicum*, and further analyzed their potential interactions with human immune proteins.

## Results

### Comparison between ES proteins and others

#### Simple sequence features

**Figure 1:**
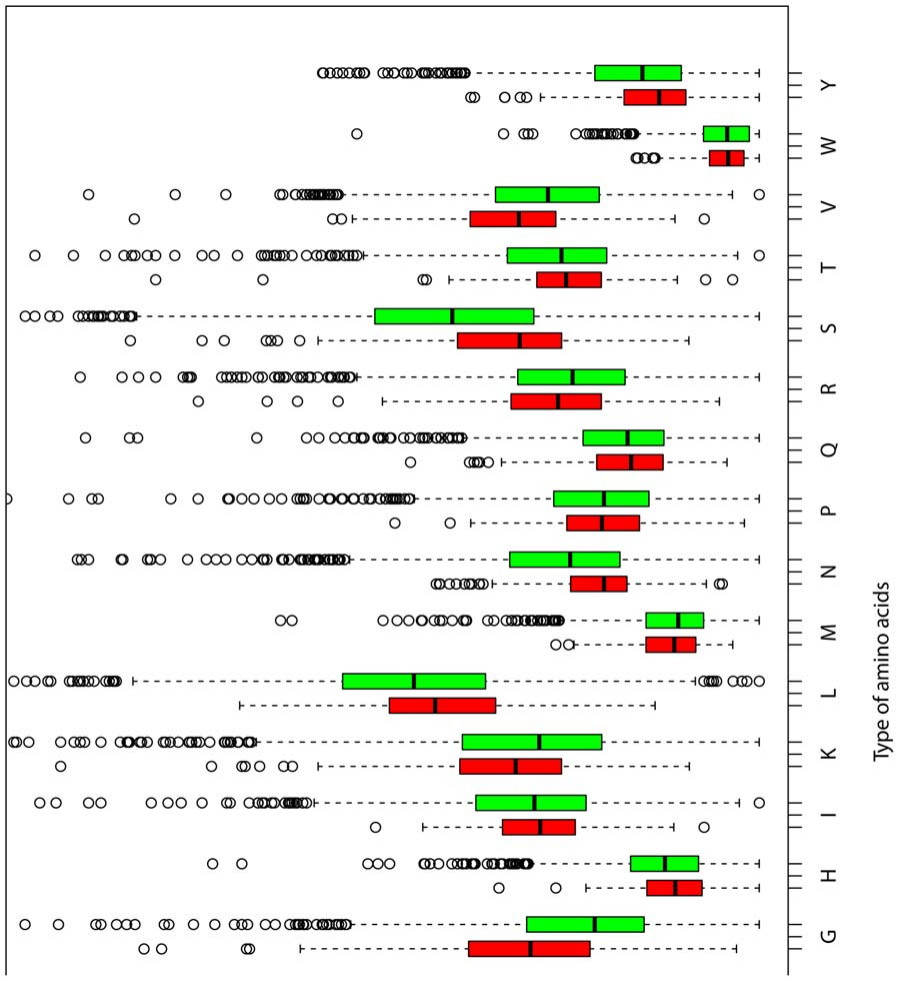
The average amino acid frequencies composition of SjSPs and SjBgPs.

**Figure 2:**
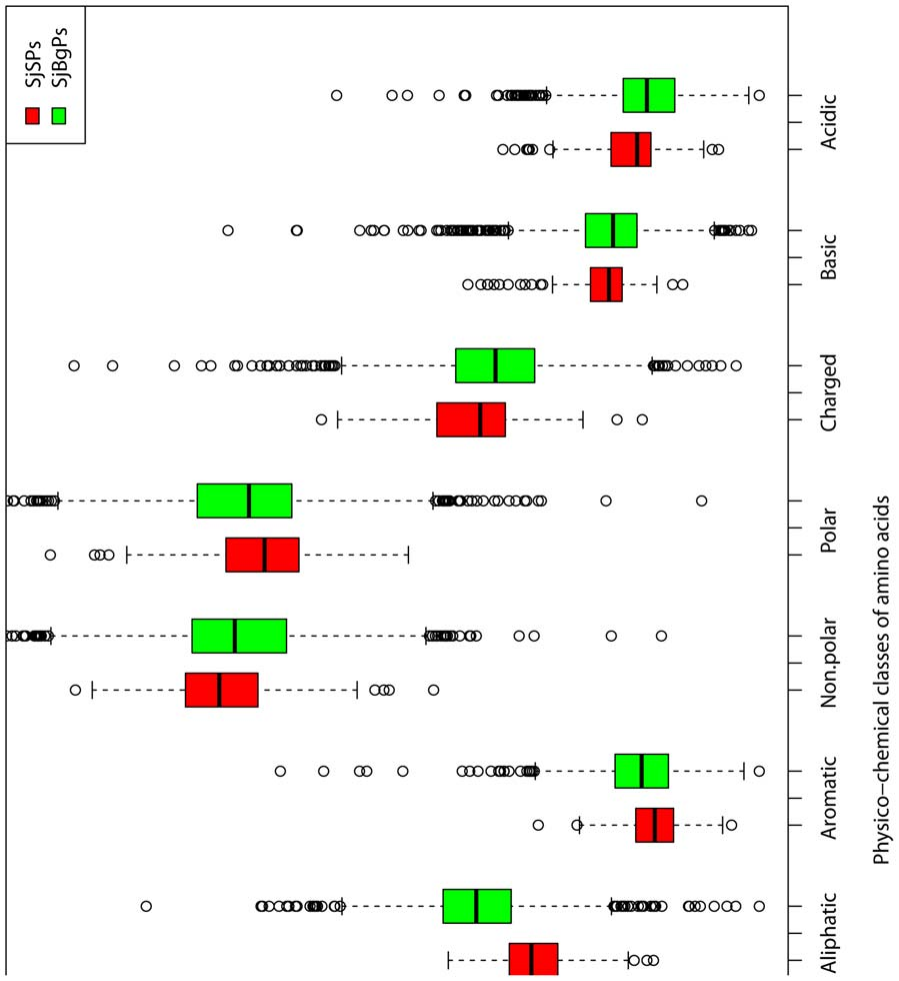
The average percentage of nine physico-chemical classes of amino acids in SjSPs and SjBgPs.

**Figure 3:**
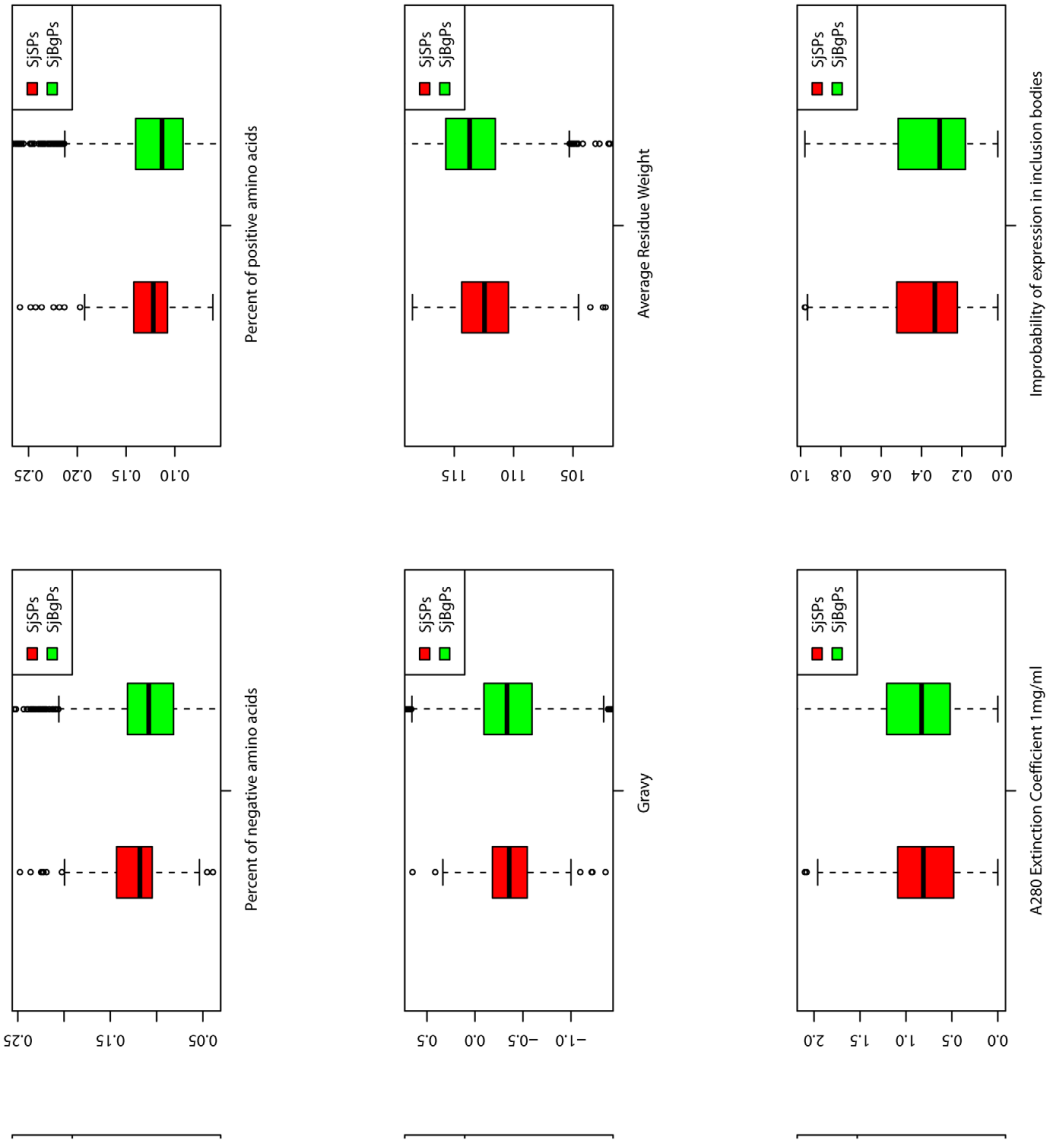
Comparison of the distributions of other primary sequence features between SjSPs and SjBgPs. Gravy: Grand average of hydropathicity. Extinction coefficient: an index indicates how much light a protein absorbs at a certain wavelength. Instability index: an estimate of the stability of your protein in a test tube.

Compared with SjBgPs, SjSPs tend to have more amino acid A, D, E, G, K and V but less N, C, H, I, L, F, S and Y (P-value <0.01, Fig. 1). Among the nine physico-chemical classes of amino acids, SjSPs have higher percentage of charged, acidic amino acids and lower percentage of aliphatic and aromatic amino acids than SjBgPs (P-value <0.01, Fig. 2). Besides, SjSPs have more amino acid number, more percent of negative (Asp+Glu) and positive amino acids (Arg+Lys), and smaller average residue weight (P-value <0.01, Fig. 3). SjSPs are more stable than SjBgPs as instability index which is used to estimate the stability of protein in a test tube of SjSPs is smaller than that of SjBgPs (P-value = 1.16e−08) and 53.3% of SjSPs are predicted as stable comparing to 37.3% of SjBgPs (P-value = 2.59e−6). Detailed comparison of all primary sequence features between SjSPs and SjBgPs are shown in Table S1.

#### Post-transcriptional modifications

**Figure 4:**
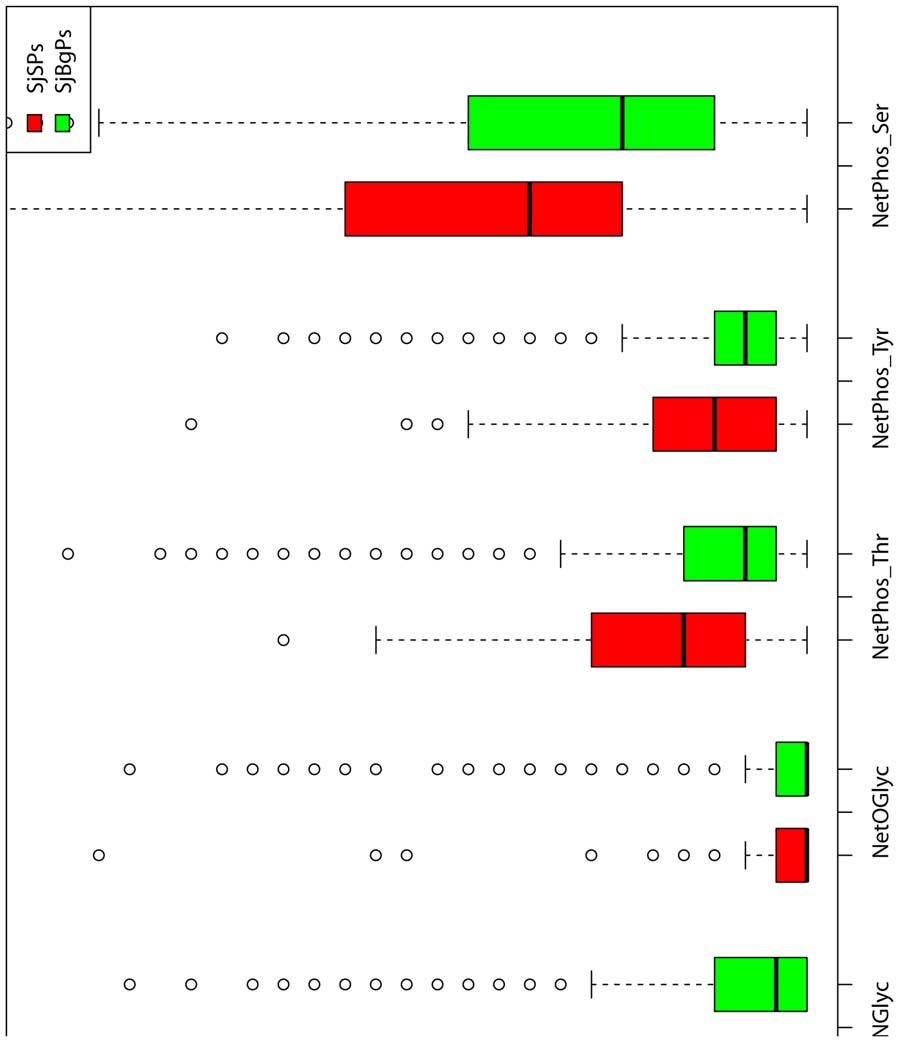
The distributions of all post-modification features of SjSPs and SjBgPs. NetCGlyc: C-mannosylation sites. NetNGlyc: N-linked glycosylation sites. NetOGlyc: O-GalNAc (mucin type) glycosylation sites. NetPhos_Ser: Ser phosphorylation sites. NetPhos_Thr: Thr phosphorylation sites. NetPhos_Tyr: Tyr phosphorylation sites.

SjSPs and SjBgPs have no difference between the percent of proteins that have C-mannosylation sites, N-linked glycosylation sites and O-GalNAc glycosylation sites (P-value >0.01, Fig. 4) but higher percent of proteins that have Ser (99.4% vs. 96.4%, P-value = 1.55e−03), Thr (96.4% vs. 83.4%, P-value = 6.34e−09) and Tyr phosphorylation sites (88.6% vs. 80.3%, P-value = 8.18e−04). As to the number of these sites for each protein, SjSPs also have more Ser, Thr and Tyr phosphorylation sites than SjBgPs (P-value = 1.56e−05, 8.90e−14, 4.90e−08 respectively). Detailed comparison can be found in Table S2.

#### Secondary structures

**Figure 5:**
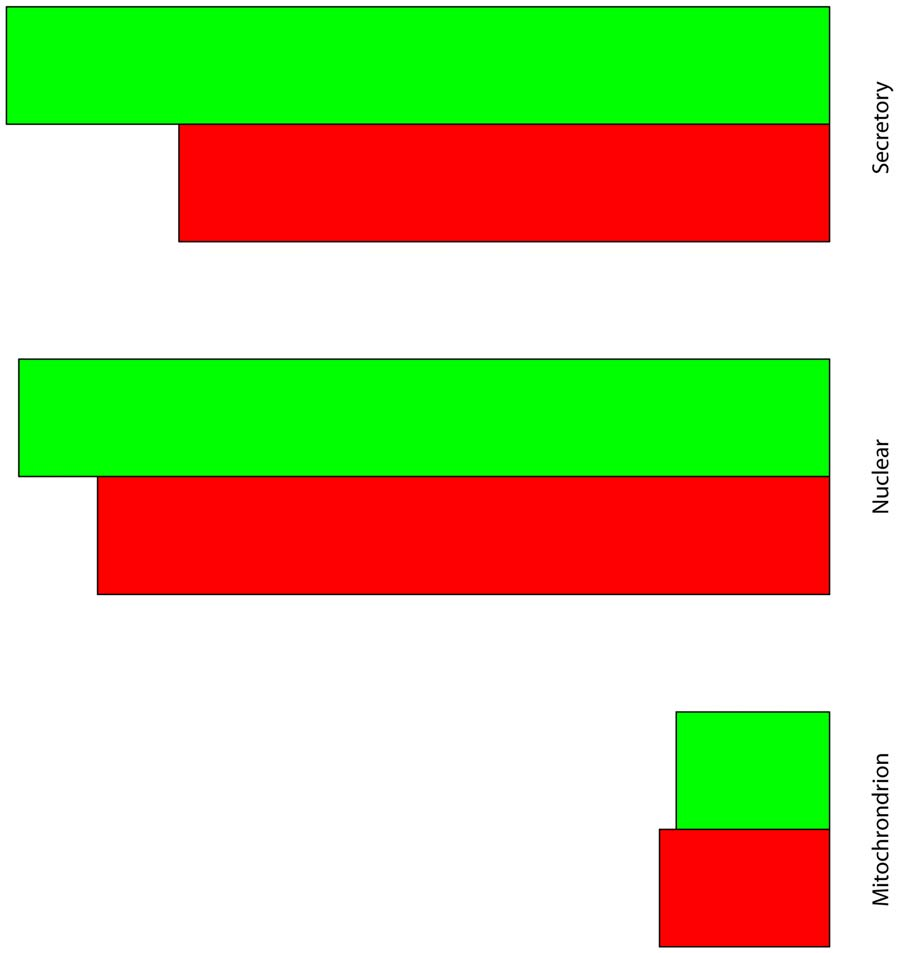
The average composition of secondary structures of SjSPs and SjBgPs.

Compared with SjBgPs, SjSPs have higher percentage of helix and coils (P-value = 5.84e−11 and 1.64e−03), and lower percentage of turns (P-value = 1.19e−06, Fig. 5). In the result of TMHMM's prediction, only 6.00% proteins of SjSPs have transmembrane regions, but 18.5% in SjBgPs (P-value = 8.22e−07). Detailed comparison can be found in Table S3.

#### Immune peptides

**Figure 6:**
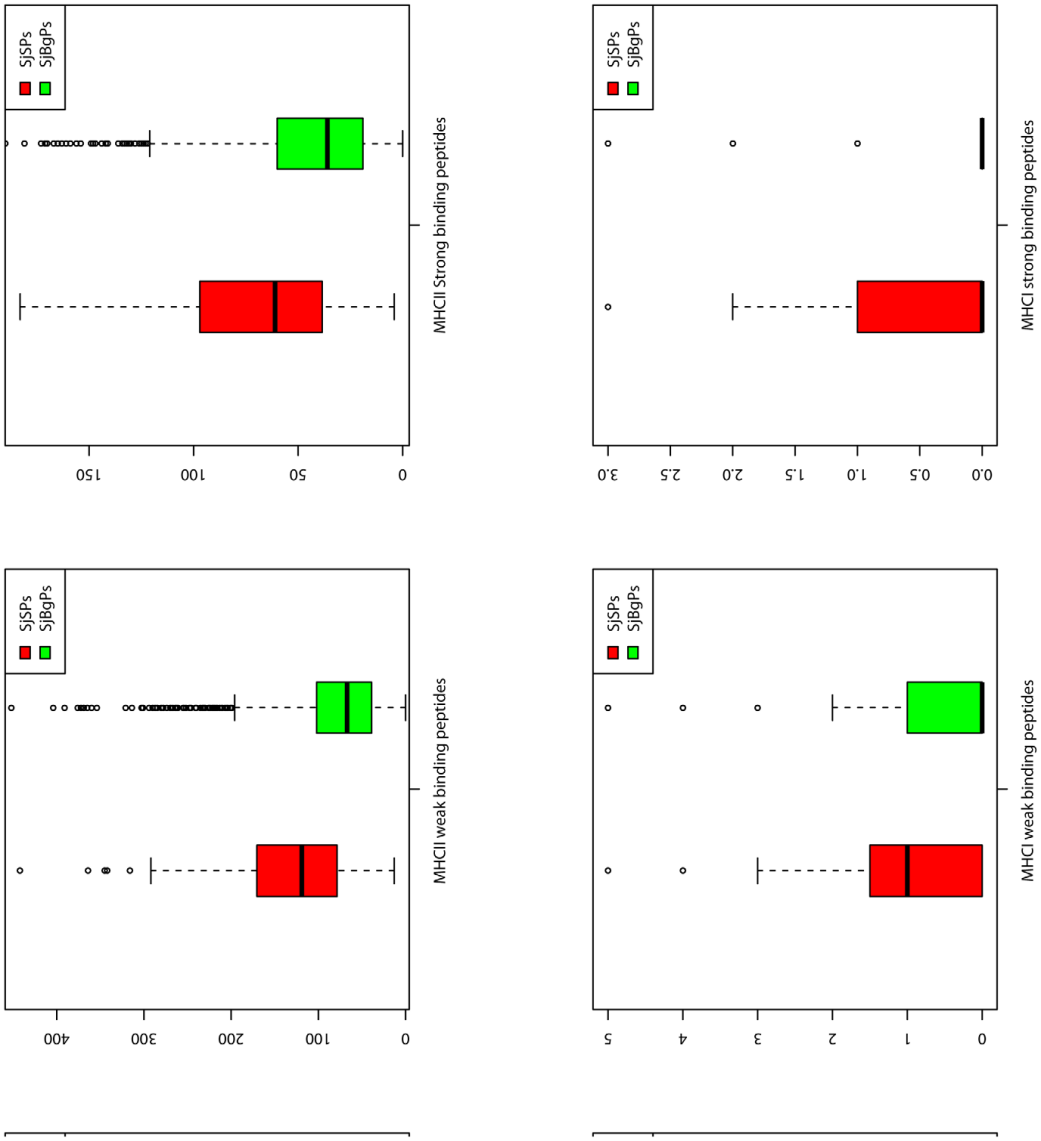
The distributions of all immune peptide features of SjSPs and SjBgPs. BepiPred: linear B-cell peptides. NetChop: Human proteasome cleave sites.

SjSPs and SjBgPs have similar percent of the proteins that have weak, strong binding peptides of MHC I, II alleles, linear B-cell epitopes and human proteasome cleave sites except the Cytotoxic T lymphocyte (CTL) epitopes (98.2% vs. 74.4%, P-value <0.01). Then, as to the number of each kind of immune peptide for each protein, we found that SjSPs had more number of weak binding peptides of MHC I and MHC II alleles (P-value = 4.78e−03 and 1.90e−16), more number of strong binding peptides of MHC II alleles (P-value = 1.76e−12), more linear B-cell epitopes (P-value = 1.01e−12), CTL epitopes (P-value = 1.07e−06) and human proteasome cleave sites (P-value = 2.60e−18) than SjBgPs (Fig. 6). These properties are consistent with ES proteins' function in immune evasion during schistosomiasis. Detailed comparison can be found in Table S4.

#### Signal peptides

**Figure 7:**
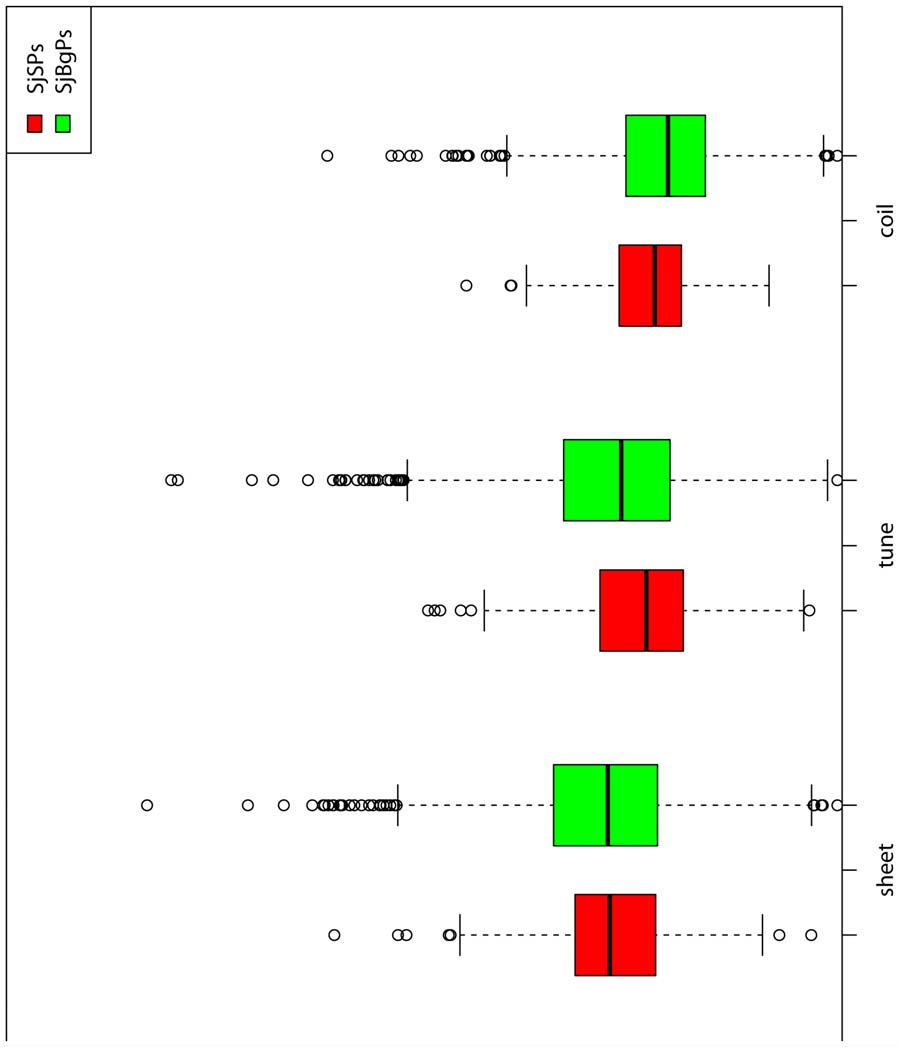
The percentage of all signal peptides in SjSPs and SjBgPs. SignalP: secretory pathway signal peptide predicted by SignalP program. Mitochondria: mitochondrial target sites. Nuclear: export nuclear target sites. Secretory: secreted via non-classical secretory pathway.

Similar percentage of SjSPs and SjBgPs have at least one mitochondrial targeting peptide (13.8% vs. 12.4% in SjBgPs), but fewer proteins in SjSPs possess secretory pathway signal peptide (11.4% vs. 17.3%, P-value = 0.016). The results are similar with the output of SecretomeP which predicts 66.7% of SjBgPs as secreted proteins while 52.7% of SjSPs (P-value = 4.58e−05, Fig. 7). In summary, distinct from mammal and bacteria only a limited number of schistosoma ES proteins contain signal peptides. Detail information of comparison is shown in Table S5.

#### Enzyme class

81.4% of ES proteins are enzymes, while only 51.0% of SjBgPs are enzymes (P-value <0.01). Within these enzymes, SjSPs contain more lyases (25.1% vs. 10.3%, P-value = 6.85e−10), isomerase (12.0% vs. 4.64%, P-value = 6.71e−06) and ligase (12.6% vs. 5.78%, P-value = 8.71e−05) than SjBgPs.

#### Domain class

31 domains with two or more *S.japonicum* ES proteins annotated are significantly enriched in SjSPs (P-value <0.01, Table S6). Three of them are related with heat shock protein 70 (IPR001023, IPR013126 and IPR018181), the members of which respond to environmental stresses and may be secreted to manipulate host's immune response in schistosomiasis [14]. Two domain are associated with thioredoxin (IPR017936 and IPR012336), whose member such as thioredoxin glutathione reductase was identified as protective antigen and drug target in Schistosomes [15], [16], [17]. Besides, other domains such as Dynein light chain (IPR001372) and calcium-binding site (IPR018247 and IPR002048) were also found significant in SjSPs. It has been reported that some proteins similar with dynein light chain are expressed in tegument and act as antigens in Schistosomes [18], [19], and the calcium-binding proteins were found to be secreted and function in host-parasite interactions in Schistosomes [20], [21].

#### GO class

55 GO terms with three or more *S.japonicum* ES proteins annotated are significantly enriched in SjSPs compared with SjBgPs (P-value<0.01, see Table S7). Among them, 23 are terms of biological processes; 14 are terms of cellular component and 18 are terms of molecular function. In the terms of biological process, most are associated with metabolic and physiological process such as DNA metabolic process (GO:0006259), dicarboxylic acid metabolic process (GO:0043648) and protein metabolic process (GO:0019538). Other terms are associated with regulation, development or stress response including regulation of growth rate (GO:0040009), negative regulation of apoptosis (GO:0043066), response to stimulus (GO:0050896) and response to stress (GO:0006950) and so on. After penetrating host, *S.japonicum* will transfer to schistosomula, and then develop into a mature stage and ultimately produce eggs. SjSPs related to metabolism, regulation and development may be important in schistosoma's maturation and response to host immune system. Besides, SjSPs are also significantly enriched in GO terms “response to drug” and “response to salt stress”, which might be important in schistosoma's fighting with drug or other stresses.

### Predicting *S.japonicum* ES proteins

In the above discussion, we find that ES proteins of *S.japonicum* do not necessarily contain secretory signal peptide and transmembrane regions, but they still have other sequence features different from background proteins. In this section, we collected 126 *S.japonicum* nuclear and histone related proteins from NCBI non-redundant proteins database as non-ES proteins of *S.japonicum* (SjnSPs). Then we randomly selected 120 proteins from SjSPs and the same number of proteins from SjnSPs, then divided them into 8 groups so that each group had 15 positive and 15 negative proteins. For each SVM-based classifier, we left a group of proteins to test the SVM accuracy and used the remaining positive and negative proteins to train. Therefore, eight SVM-based classifiers were generated in total. The average accuracies were 81.7% (sensitivity) and 84.2% (specificity) for positive and negative dataset respectively. We defined that protein was predicted as ES protein only when all eight SVM classifiers predicted it as ES protein. By using the above strategy, the final precision to predict ES proteins was 100% for 8 groups of tested proteins and the average recall is 81.7%, which is much higher than state-of-art methods: SignalP [12], SecretomeP [13], Phobius [22] and ProtComp [23] (Table 1). Using our classifier, we identified 242 SjBgPs as potential ES proteins from SjBgPs (Table S8). Among them, 51 (21.1%) proteins have already been reported as ES proteins or locating in tegument of Schistosomes (Table S8). For example, *glutathione S-transferase* (CAX72404.1) can stimulate anti-fecundity immunity [24]. *Thioredoxin peroxidase* (|CAX75864.1), an egg secretory product and antioxidant enzyme, is necessary for schistosome to escape oxidative damage from host immune system [25], [26]. Another important protein is *paramyosin* (ACA62791.1) which is released by schistosome cercariae while penetrating human skin. It may function in immune evasion and immune response during infection [27], [28]. Furthermore, *tetraspanin* (CAX70119.1) expresses in the tegument and functions in the host immune response and invasion. It was suggested that tetraspanin might interact with host ligand such as MHC and was considered as a candidate vaccine for schistosome [29], [30]. *Fatty acid binding protein 7* (CAX77287.1) is secreted by adult *S.japonicum* and plays an important role in the uptake and transport of host-derived fatty acids for the schistosomes lacking the system of fatty acids synthesis [10]. In addition, *superoxide dismutase* (CAX71766.1) is also reported as ES protein [21]. All these facts suggest the accuracy of our SVM classifier.

**Table 1:** Comparsion of prediction accuracy for ES proteins of Schistosoma japonicum.

	Phobius	ProtComp	SecretomP	SignalP	SVM
**precision**	77.8%	52.0%	52.7%	76.0%	100%
**recall**	8.40%	7.80%	52.7%	11.4%	81.7%

The precision and recall of Phobius, ProtComp, SecretomP and SignalP are evaluated by the dataset of SjSPs and SjnSPs. The performance of SVM is evaluated by the eight groups of tested proteins.

### Predict host-*schistosoma japonicum* interaction

**Figure 8:**
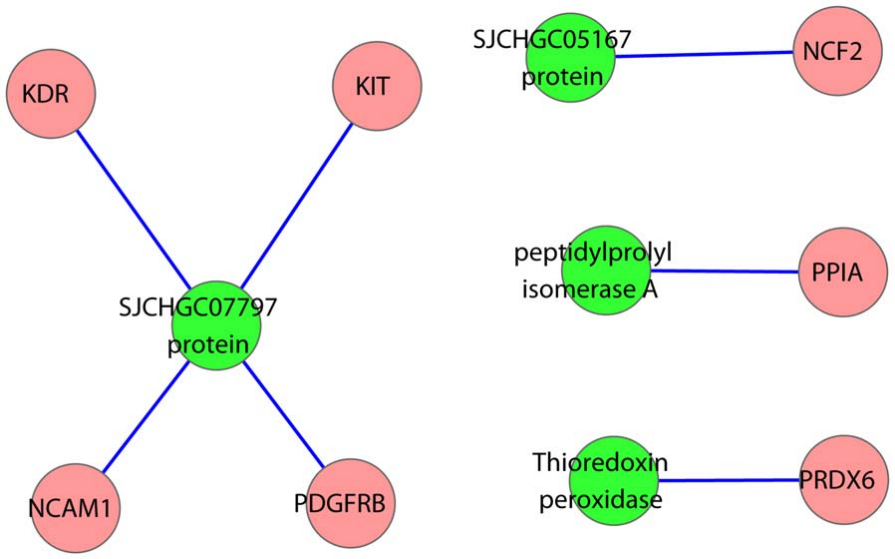
Potential protein protein interactions between Schistosoma japonicum and human.

Proteins secreted by *S.japonicum* were considered to be involved in host-parasite interaction [9]. In our analysis, 7 interactions between 4 ES proteins of *S.japonicum* and 7 human immune proteins were identified following the pipeline described in Methods (Table S9 and Fig. 8). Among the 4 ES proteins, *SJCHGC07797* protein (AAX30806.1)has the largest number of interacting partners (4 proteins, Fig. 8). One of the partners, called *neural cell adhesion molecule 1* (*NCAM1*), is involve in the expansion of T cells and dendritic cells which play an important role in immune surveillance in human. *Neural cell adhesion molecule* was found to be upregulated in Chagas' disease myocarditis which caused by the *Trypanosoma cruzi* (*T. cruzi*) parasite and considered as a receptor for tissue targeting and cellular invasion by *T. cruzi* in Chagas' disease [31]. Another partner named platelet-derived growth factor receptor beta precursor (*Pdgfrb*) was upregulated in liver of human infected by *Clonorchis sinensis* [32]. Another ES protein *Thioredoxin peroxidise* was predicted to interact with peroxiredoxin 6 (*PRDX6*), which is important for host defence against *Opisthorchis viverrini* infection [33]. The functions of these human proteins are associated with parasite interaction while other parasites infected, suggesting the similar function against to *S.japanicum*.

## Discussion

Compared with other proteins, ES proteins are generally longer, more likely to be stable and enzyme, more likely to contain immune-related binding peptides and more likely to be involved in regulation and metabolism processes. However, only a limited number of schistosoma ES proteins contain signal peptides, leading to the failure of popular prediction tools e.g. SignalP. In this paper, we combined 1,067 sequence features and designed a SVM-based classifier to distinguish ES proteins from other proteins of *S.japonicum*. Using this SVM, we identified 242 potential ES proteins, among which 51 are known previously. According to the prediction, the ES proteins are taken up 17.3% of the whole protein set for prediction. The relatively high percentage of ES proteins in *S.japonicum* suggests the importance of them in schistosoma infection.

Long term survival of schistosomes relies on their interactions with their host, which resulting in the escape of immune response and induction of immunological tolerance. ES proteins may mimic host proteins and directly or indirectly to modulate host immune response [34]. However, the molecular mechanisms involved in the host-parasite interactions are still poorly understood. We further identified 7 potential interactions between ES proteins and human immune proteins based on DDI and sequence similarity. These interactions may help reveal the pathogenesis of schistosomiasis and find out new vaccines and drug targets.

## Materials and Methods

### Dataset

We collected excretory/secretory proteins of *S.japonicum* including tegument proteins that are exposed to the host from public literatures [9], , denoted as SjSPs. As control, we randomly selected 2,411 proteins (about 10%) out of all NCBI-recorded *S.japonicum* proteins as background, denoted as SjBgPs. We used PISCES [35] to remove proteins with sequences identity over 20% in each dataset. Then, to obtain the dataset of SjSPs more accurately, we further filtered the ES proteins with expression level in hepatic schistosomulum, adult and egg lower than 1 [9]. Finally, we got 167 SjSPs and 1,401 SjBgPs. To get a negative dataset for SVM training, we collected the nuclear and histone related proteins from NCBI non-redundant database and also remove proteins with sequences identity over 20% by PISCES [35], resulting in 126 negative proteins (non-ES, denoted as SjnSPs).

### Sequence features of proteins

#### Primary sequence features

Pepstats [36] was used to calculate average residue weight, isoelectric point, probability of protein expression in E. coli inclusion bodies, extinction coefficient at 1 mg/ml (A280) and 9 physico-chemical classes of amino acids including tiny (A, C, G, S and T); small (A, B, C, D, G, N, P, S, T and V); aromatic (F, H, W and Y); non-polar (A, C, F, G, I, L, M, P, V, W and Y); polar (D, E, H, K, N, Q, R, S, T and Z), charged (B, D, E, H, K, R, Z), aliphatic (A, I, L, V) and basic (H, K, and R). ProtParam [37] were used to calculate the basic parameters including the composition of each amino acid, total number of amino acid, molecular weight, percent of negatively or positively charged residues, estimated half-life and instability index.

#### Structure properties

The percentage of four secondary structure types (alpha helix, beta sheet, turns and coil) were calculated using garnier [38]. TMHMM [39], [40] was used to predict the number of transmembrane helical regions.

#### Immunology peptides

As ES proteins of *S.japonicum* play a key role in immune response, we counted the number of several immunology peptides including MHC class I weak and strong peptides [41], MHC class II weak and strong peptides, linear B-cell peptides [42], human proteasome cleave sites [43] and cytotoxic T-lymphocyte epitope (CTL) epitopes [44] using tools listed in Table S10.

#### Target and signal sites

Mitochondria target sites [45], export nuclear target sites [46], signal peptide cleavage sites [12] and secreted signal sites [13] were predicted using tools listed in Table S11.

#### Post-translational modification

We analyzed several post-translational modifications *i.e.* C-mannosylation, N-glycosylation [47], O-glycosylation [48], N-terminal acetylation sites and Try, Thr, Ser phosphorylation sites [49] using tools listed in Table S12.

#### Enzyme classes

ProtFun [50] was used to predict whether a protein is an enzyme, and which category (oxidoreductase, transferase, hydrolase, lyase, isomerase and ligase) it belongs to.

#### Domain analysis of proteins

Domain information of each protein was parsed using InterProScan [51], a web server that combines multiple methods to search against InterPro database through sequence similarity.

#### Gene Ontology

The GO of SjSPs, SjnSPs and SjBgPs were annotated using Blast2GO with default parameter settings [52].

#### Statistical test

Sided t test was used to evaluate the difference between the means of each feature with continue value, while for the features with boolean values, hypergeometric test was used to evaluate whether the proteins with the feature are significantly enriched or less in SjSPs compared with SjBgPs.

### SVM and ES protein prediction

The basic concept of SVM is to transform the input vectors to a higher dimensional space through kernel functions, and then a hyperplane that maximizes the margin between positive and negative samples is found [53]. The SVM for ES protein classification depends on the selection of kernel function. Because of its localized and finite responses across the entire range of the real x-axis, radial basis function (RBF) is by far the most popular choice of kernel used in SVM. In this study, we also used SVM with RBF kernel to train features of ES proteins.

167 SjSPs and 126 SjnSPs were used as positive and negative dataset respectively in the training. After removing features that are missed in over 10% of the proteins, 1,067 sequence features were used to train the SVM. All features are scaled across training and testing samples to zero mean and unit variance. The objective is to avoid features in greater numeric ranges dominate those in smaller numeric ranges.

We randomly selected 120 proteins from 167 positives and other 120 proteins from 126 negatives. Then we divided these proteins into 8 groups so that each group had 15 positive and negative proteins. For each SVM classifier, we left a group of proteins to test the SVM accuracy and used the remaining positive and negative proteins to train. To find the best model, we searched through a large range of the cost parameter and gamma parameter in RBF kernel with 10-fold cross validation. In total, eight SVM classifiers were generated. Finally, we predicted the SjBgPs dataset by 8 classifiers and selected those proteins that were predicted as “true” by all classifiers. The SVM training was done by “e1071” R package.

### Prediction of host-parasite interactions

We only considered host-parasite interactions between human immune system related proteins and *S.japonicum* ES proteins. It has been reported that schistosoma ES proteins may mimic human protein with similar sequence or domains, and hence evade or regulate immune response [54]. Therefore we made our predictions based on protein domain-domain interaction (DDI) and sequence similarity. More specifically, a human protein and a *S.japonicum* ES protein is considered to interact with each other if i) their domains could form at least one known DDI, ii) this ES protein is similar to at least one known interacting partner of the human protein. Our threshold of similarity is aligned length > = 30 and identity > = 30% by BLAST. Human immune system related proteins were downloaded from Immunome database[55] and human protein-protein interactions from HPRD [56]. Besides, we collected the domain-domain interactions that are inferred from PDB [57] from the DOMINE database [58], which contains both interactions inferred from PDB and predicted from different computational approaches.

## Supporting Information

Table S1 Detail comparison of primary features between SjSPs and SjBgPs.
(XLS)

Table S2 Detail comparison of post-transcriptional modifications between SjSPs and SjBgPs.
(XLS)

Table S3 Detail comparison of secondary features between SjSPs and SjBgPs.
(XLS)

Table S4 Detail comparison of immune peptides between SjSPs and SjBgPs.
(XLS)

Table S5 Detail comparison of signal peptides between SjSPs and SjBgPs.
(XLS)

Table S6 Domains significantly enriched in SjSPs against SjBgPs by hypergeometric test.
(XLS)

Table S7 Significantly enriched GO terms in SjSPs against SjBgPs by hypergeometric test.
(XLS)

Table S8 Predicted ES proteins of S.japonicum by SVM.
(XLS)

Table S9 Predicted host-parasite interactions between human and S.japonicum.
(XLS)

Table S10 Tools used to predict immunology peptides.
(XLS)

Table S11 Tools used to predict target sites and signal peptides.
(XLS)

Table S12 Tools used to predict post-translational modifications.
(XLS)
